# Inhibition of Xenograft Tumor Growth by Gold Nanoparticle-DNA Oligonucleotide Conjugates-Assisted Delivery of *BAX* mRNA

**DOI:** 10.1371/journal.pone.0075369

**Published:** 2013-09-20

**Authors:** Ji-Hyun Yeom, Sang-Mi Ryou, Miae Won, Mira Park, Jeehyeon Bae, Kangseok Lee

**Affiliations:** 1 Department of Life Science, Chung-Ang University, Seoul, Republic of Korea; 2 Department of Pharmacy, CHA University, Seongnam, Republic of Korea; 3 College of Pharmacy, Chung-Ang University, Seoul, Republic of Korea; Center for Genomic Regulation, Spain

## Abstract

Use of non-biological agents for mRNA delivery into living systems in order to induce heterologous expression of functional proteins may provide more advantages than the use of DNA and/or biological vectors for delivery. However, the low efficiency of mRNA delivery into live animals, using non-biological systems, has hampered the use of mRNA as a therapeutic molecule. Here, we show that gold nanoparticle-DNA oligonucleotide (AuNP-DNA) conjugates can serve as universal vehicles for more efficient delivery of mRNA into human cells, as well as into xenograft tumors generated in mice. Injections of *BAX* mRNA loaded on AuNP-DNA conjugates into xenograft tumors resulted in highly efficient mRNA delivery. The delivered mRNA directed the efficient production of biologically functional BAX protein, a pro-apoptotic factor, consequently inhibiting tumor growth. These results demonstrate that mRNA delivery by AuNP-DNA conjugates can serve as a new platform for the development of safe and efficient gene therapy.

## Introduction

Messenger RNA (mRNA) is a promising vehicle for expression of target proteins in both the treatment of genetic diseases and for vaccination (for recent reviews, see 1-3). Compared to plasmid DNA (pDNA)-mediated gene delivery, mRNA-based transfection confers several advantages. First, mRNA delivery is safer than pDNA delivery, as mRNA does not integrate into the genome, leaving the transfection transient. Second, mRNA does not need to enter the nucleus to perform its function, whereas in pDNA delivery, the nuclear membrane presents as a major barrier. Moreover, transcriptional initiators (promoters) and terminators are dispensable for synthetic mRNAs, making them relatively easy to engineer for therapeutic molecules. Taken together, these features demonstrate the potential for mRNA use in gene therapy.

Various delivery systems for mRNA have been developed and can be classified into two main groups: biological and non-biological agent-based systems. Biological agent-based systems utilize mostly viral vectors. Although they exhibit high transfection efficiency, they also trigger immune responses and integration of viral genes into the host genome. In contrast, non-biological systems are non-pathogenic and relatively safe, but clinical applications are obstructed by transfection efficiency. To overcome this problem, numerous non-biological agents based on cationic lipids [4], polyethylenimine [5], poly-L-lysine [6], and dendrimers [7] have been developed. Among these systems, liposome-based materials appear to exhibit especially high-efficiency mRNA transfection and protein expression in cultured cells [8-12]. Lipofectamine, a commonly used and commercially available liposomal formulation, has high transfection efficiency for both DNA and mRNA cell delivery. However, in delivering nucleic acids in vivo into animals, liposome proved ineffective and caused immunological responses [13,14]. Further, liposome-based systems cause significant cytotoxicity, as well as rapid clearance of DNA or mRNA due to overcharged liposome complexes interacting with serum proteins [15-17]. For these reasons, the development of a safe and efficient method of mRNA delivery into living systems has been challenging, despite the advantages of using mRNA over other biomaterials as a therapeutic molecule.

We have previously demonstrated that gold nanoparticles (AuNP) conjugated to a universal cargo DNA can be a convenient and efficient method of delivering DNA oligonucleotides and small RNAs, including shRNA and aptamers, into living cells; this can be achieved without causing significant cytotoxicity, even during long incubation periods (1–5 days) [18-21]. Such properties are advantageous in biomedical applications of gene delivery systems, which require careful safety measures (for recent reviews, see 22,23). We thus investigated in this study the potential of AuNP-DNA conjugates to deliver mRNA. Our results demonstrate that mRNA delivered by these conjugates can direct successful synthesis of biologically functional proteins in living systems, providing a novel and efficient carrier for mRNA delivery.

## Materials and Methods

### Ethics statement

These studies were carried out in accordance with an animal protocol approved by the Chung-Ang University Support Center for Animal Experiments. Animals were sacrificed using overdoses of bicarbonate gases, and all efforts were made to minimize suffering.

### Synthesis of AuNP-DNA conjugates

Standard citrate-reduced AuNP (15 nm diameter) were purchased from BBI Life Science (UK). DNA oligonucleotides (αRNA I) were conjugated to AuNP according to previously described procedures [24]. Briefly, thiolated RNA I oligonucleotides (oligo) were treated with 1N dithiothreitol (DTT) for 30 min at room temperature (RT) to cleave the disulfide bond. Excess DTT and unwanted thiol fragments from the free thiol-modified oligonucleotide mixture were removed by extracting three times with ethyl acetate. The cleaved oligonucleotides were purified using an ethanol precipitation method. AuNP were mixed with oligonucleotides at a ratio of 1:100, and citrate-HCl buffer (0.5 M, pH 3) added to achieve a final concentration of ~10 mM. After briefly vortexing, the sample was incubated at RT for 3 min. Next, the pH of the AuNP solution was adjusted to neutral using 0.5 M HEPES buffer (pH 7.6). Following further incubation at RT for 5–10 min, the AuNP-Oligo mixture was centrifuged at 13,000 × *g*, and the supernatant was removed. The final mixture was re-dispersed in 5 mM HEPES buffer. The concentration of functionalized AuNP was determined by UV-Vis spectroscopy. The absorbance values were used to measure the nanoparticle concentration via Beer’s law (A= εbc). The wavelength of the absorbance maximums (λ) and extinction coefficients (ε) used for 15 nm particle size are as follows: λ = 524 nm, ε = 2.4 ×10^8^ L/(mol·cm).

### *In vitro* synthesis of mRNA

The mRNA molecules were synthesized from DNA templates containing a T7 promoter followed by a sequence of *dsRED*, *GFP*, and *BAX* using the MEGAscript^TM^ kit (Ambion, USA), according to the manufacturer’s instructions. *In vitro* synthesized mRNA molecules were comprised of 5′-end Kozak sequences (5′-GCCGCCACC-3′), an open reading frame (ORF) beginning with a start codon and ending with a stop codon, and a polyA tail consisting of 20 nucleotides (ntds) at the 3′-end. DNA templates were prepared using PCR, with primers purchased from Bioneer Corporation (Korea), as listed in Table 1. pdsRED, pEGFP-C1, and pcDNA3-BAX vector were used as templates to amplify DNA segments containing the coding region for dsRED, GFP, and BAX, respectively. The 5′-end capped transcripts were synthesized using the cap analog [m^7^G(5′) ppp(5′) G] incorporated by mMESSAGE mMACHINE kit (Ambion, USA). All mRNAs contained a sequence complementary to RNA I oligo, which hybridizes to AuNP-αRNA I [18]. The synthesized mRNA molecules were purified using phenol extraction or Illustra^TM^ MicroSpin^TM^ G-50 columns, according to the manufacturer’s instructions (GE, UK).

**Table 1:** PCR primers used for synthesis of mRNA.

PCR primer	Nucleotide sequence (5' to 3')	mRNA synthesized
5′RNAI-dsRED-F	CTTAATACGACTCACTATAGGGCGCTAGCAGAGCCGAGATGCCGCCACCATGGCCTCCTCCGAGGACG	5′dsRED mRNA and 5′, 3′dsRED mRNA
5′RNAI-dsRED-R	TTTTTTTTTTTTTTTTTTTTCTACAGGAACAGGTG	5′dsRED mRNA and 5′∆RNAI-dsRED mRNA
3′RNAI-dsRED-F	CTTAATACGACTCACTATAGGGGCCGCCACCATGGCCTCCTCCGAGGACG	3′dsRED
3′RNAI-dsRED-R	TTTTTTTTTTTTTTTTTTTTCGCTAGCAGAGCCGAGATCTACAGGAACAGGTGGT	3′dsRED and 5′, 3′dsRED mRNA
5′∆RNAI-dsRED-F	CTTAATACGACTCACTATAGGGGCCGCCACCATGGCCTCCTCCGAGGACG	5′∆RNAI-dsRED mRNA
5′RNAI-GFP-F	TAATACGACTCACTATAGGGCGCTAGCAGAGCCGAGATTACCGGTCGCCACCATGGTGAGCA	5′GFP mRNA
5′RNAI-GFP-R	TTTTTTTTTTTTTTTTTTTTTTATCTAGATCCGGTGGATCCCGGGCC	5′GFP mRNA
5′RNAI-BAX-F	CTTAATACGACTCACTATAGGGCGCTAGCAGAGCCGAGATGCCGCCACCATGGACGGGTCCGGG	5′BAX mRNA
5′RNAI-BAX-R	TTTTTTTTTTTTTTTTTTTTTCAGCCCATCTTCTTCCA	5′BAX mRNA and 5′BAX-null mRNA
5′RNAI-BAXnull-F	CTTAATACGACTCACTATAGGGCGCTAGCAGAGCCGAGATGCCGCCACCTAAGACGGGTCCGGG	5′BAX-null mRNA
RNA I-AUG	CGCTAGCAGAGCCGAGATGCCGCCACCATG	RT-PCR of *BAX* mRNA
BAX 3’	TTCTCGAGTCAGCCCATCTTCTTCCAGAT	RT-PCR of *BAX* mRNA
GAPDH 5’	AGCCAAAAGGGTCATCATCTCT	RT-PCR of *GAPDH* mRNA
GAPDH 3’	AGGGGCCATCCACAGTCTT	RT-PCR of *GAPDH* mRNA

### Preparation of the AuNP-DNA-mRNA complex

The mRNA was pre-incubated at 80°C for 5 min and quickly chilled on ice to prevent secondary structure formation. AuNP-αRNA I (10 nM) and mRNA (4 µM, unless otherwise indicated) were annealed for 10 min at 55°C in 1× phosphate-buffered saline (PBS) containing 0.3 M NaCl and stored at 4°C. The resulting conjugates were spun at 13,000 × *g* for 10 min, the supernatant removed, and the conjugate pellet re-suspended in PBS. These precipitation and re-suspension steps were performed three times.

### Mammalian cell culture, delivery of functionalized AuNP-αRNA I-mRNAs, and liposome transfection

Human cervical carcinoma (HeLa) cells were cultured in Dulbecco’s modified Eagle’s medium (DMEM) containing 10% heat-inactivated fetal bovine serum (FBS) and 1% penicillin-streptomycin (Welgene, Seoul, Korea). HeLa (2.5 × 10^5^) cells were grown on 10-mm lysine coated discs, incubated with AuNP*-*αRNA I-mRNA conjugates (1 nM) in the culture media for 12 h, and then fixed for experiments. For the liposome-based delivery, the cells were transfected with mRNA using Lipofectamine 2000 following manufacturer’s instructions (Invitrogen, USA).

### Measurement of mRNA loading capacity on AuNP-αRNA I

AuNP-αRNA I (10 nM) was hybridized with increasing concentrations of mRNA (2, 4, and 8 µM), and the resulting conjugates were analyzed in a 6% polyacrylamide gel containing 8 M urea. The bands were visualized with ethidium bromide and quantitated using the Quantity One Image program (Bio-Rad Laboratories, Inc., USA).

### Detection of delivery and expression of mRNA

To detect the delivery of mRNA into HeLa cells, mRNA encoding dsRED and BAX were labeled with Cy3 using the Silencer ^®^siRNA Labeling Kit, according to the manufacturer’s instructions (Ambion, USA). The AuNP-αRNA I-cy3-dsRED mRNA was incubated with cells for 12 h, and the resulting samples were fixed with 4% paraformaldehyde (Sigma, USA). The Cy3 (647 nm excitation, 670 nm emission) and dsRED (570 nm excitation, 590 nm emission) fluorescence were detected by laser scanning confocal microscopy (Carl Zeiss ZEN 2011). Relative intensities of fluorescence were measured using ImageJ software.

### Cell viability assay

HeLa cells were seeded in 96-well plates and cultured overnight to allow for cell attachment. Cells were then incubated with AuNP-αRNA I (1 nM) and increasing concentrations of *BAX* mRNA (0.1–0.4 µM). Cell viability was measured 24 h after incubation using the ATP-dependent CellTiter-Glo luminescent reagent, according to the manufacturer’s instructions (Promega Corp, USA). The absorbance was measured using a PerkinElmer 1420 Multilabel Counter (PerkinElmer, USA), and values were normalized against those for control cells. Experiments were performed in triplicate and repeated at least three times.

### Semi-quantitative RT-PCR analysis

Total RNA was extracted from the cell lines with TRI Reagent Solution (Ambion, USA), according to the manufacturer’s instructions. Synthesis of cDNA was performed with 1 µg of the total RNA using the PrimScript^TM^ 1st-Strand cDNA Synthesis kit (Takara, Japan). PCR was carried out in a total volume of 10 µl using 2 µl of RT reaction. All PCR products were fractionated using 1.2% agarose gel electrophoresis and visualized using ethidium bromide. Equal loading was ensured by using an internal control (GAPDH). Primers used are listed in Table 1. The bands were quantitated using the Quantity One Image program (Bio-Rad Laboratories, Inc., USA).

### Animal studies

BALB/c nu/nu immunodeficient mice (6 weeks old, 18–20 g) were purchased from Central Lab, Animal Inc. (Seoul, Korea). Once HeLa cells had formed a monolayer, a single-cell suspension was prepared using the trypsin digestion method. The cell number was adjusted to 1 × 10^7^ in 100 µl of PBS, and the cells were injected subcutaneously into the abdominal walls of the mice. Once the HeLa cells formed tumors (tumor volume: ~0.1 cm^3^), AuNP-αRNA I-5′ *BAX* mRNA conjugates (5 nM AuNP, 0.4 µM mRNA, one injection per day every three days) were injected directly into the tumors [20]. The long and short axial lengths of the tumors were measured every other day. Mice were killed 30 days after the first injection of the conjugates, after which the tumor samples were collected for further analyses.

### Statistical analysis

Multiple comparison analyses of values were performed with the Student-Newman-Keuls test (SAS), and the significance of the treatment values was analyzed by comparing with control values using Student’s *t*-test. The data represent the mean ± SEM, and *P* < 0.05 was considered statistically significant. The *P* values of significant results are shown.

## Results

### Delivery of mRNA into human cells by AuNP-DNA conjugates and protein expression

To test whether AuNP-DNA conjugates can be used to delivery mRNA, we synthesized *dsRED* mRNA using *in vitro* transcription. The 5′-terminus of the synthetic *dsRED* RNA contained an 18-nucleotide (ntd) sequence that was complementary to αRNA I oligo, followed by a Kozak sequence (5′-GCCGCCACC-3′), a coding region for dsRED, and a polyA tail (20 ntds). This synthetic RNA was designated as 5′dsRED mRNA. We also synthesized a derivative of 5′dsRED mRNA that did not contain an 18-nucleotide (ntd) sequence complementary to αRNA I oligo, and designated this as 5′∆RNA I-dsRED mRNA. This RNA was used to test whether loading of AuNP-αRNA I with mRNA is dependent on interactions between αRNA I oligo and its complementary mRNA sequence.

**Figure 1:**
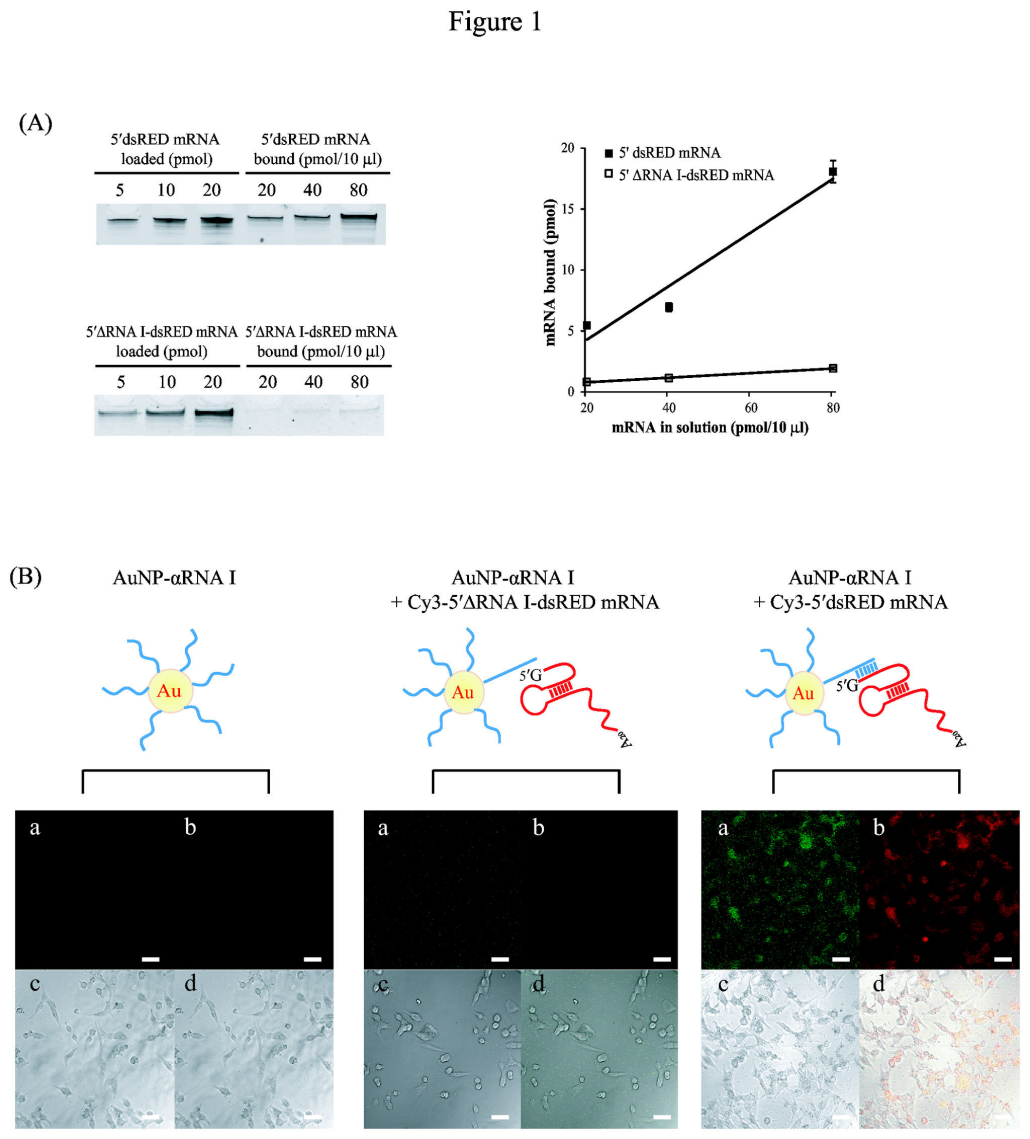
Delivery of *dsRED* mRNA to HeLa cells by AuNP-DNA conjugates. (A) Measurement of the loading capacity of AuNP-αRNA I for 5′dsRED mRNA and 5′∆RNA I-dsRED mRNA. Known amounts of 5’ dsRED mRNA or 5′∆RNA I-dsRED mRNA (5, 10, and 20 pmol) and ten µl of varying concentrations (2, 4, and 8 µM) of these mRNAs hybridized to AuNP-DNA were loaded (lanes 4-6) to assess the loading capacity of for 5’ dsRED mRNA containing a sequence complementary to αRNA I at the 5′-terminus. The concentration of AuNP-DNA was 10 nM. Amounts of mRNA bound to AuNP-DNA at each concentration were plotted and shown in the graph. (B) Efficient delivery of AuNP-αRNA I-Cy3-5 ′dsRED mRNA into HeLa cells. Cells were treated with AuNP-αRNA I alone, AuNP-αRNA I-Cy3-5’∆RNA I-dsRED mRNA, or AuNP-αRNA I-Cy3-5’dsRED mRNA; these are represented schematically in diagrams. The final concentrations of AuNP-αRNA I and dsRED mRNA were 1 nM and 0.2 µM, respectively. Representative confocal fluorescence microscopy images of HeLa cells detecting Cy3-5’dsRED and Cy3-5’∆RNA I-dsRED mRNA (a) and dsRED protein (b) are shown. Transmission images of cells (c) and overlay images of a, b, and c (d) are also shown. Bar indicates 20 µm.

We first assessed the loading capacity of AuNP-αRNA I for 5′dsRED mRNA. AuNP-αRNA I (10 nM) was hybridized with increasing concentrations (2, 4, and 8 µM) of 5′dsRED mRNA, and the resulting conjugates were analyzed on a 6% polyacrylamide gel containing 8 M urea. To quantify the amount of 5′dsRED mRNA that was bound to AuNP-αRNA I (lanes 4–6), known amounts of 5′dsRED mRNA were loaded in the first three lanes. Consistent with previously reported results [20,21], our results showed that approximately one-fourth of the 5′dsRED mRNA in the reaction mixtures were loaded onto AuNP-αRNA I at saturating concentrations of mRNA (Figure 1A, left upper panel). In contrast, 5′∆RNA I-dsRED mRNA could not be loaded onto AuNP-αRNA I at saturating concentrations of mRNA, indicating that the loading of 5′dsRED mRNA is mediated by base-pairing interaction between αRNA I oligo and its complementary sequence in 5′dsRED mRNA (Figure 1A, left low panel).

Next, to examine whether AuNP-αRNA I could deliver mRNA into living systems, we applied AuNP-αRNA I loaded with Cy3-labeled 5′dsRED mRNA (AuNP-αRNA I-Cy3-5′dsRED mRNA) to HeLa cells. The efficiency of mRNA delivery was assessed by visualizing dsRED mRNA using confocal microscopy 12 h after this treatment. The Cy3 fluorescence signal was uniformly detected in cells treated with AuNP-αRNA I-Cy3-5′dsRED mRNA, while no fluorescence was detected in cells treated with AuNP-αRNA I alone or a mixture of AuNP-αRNA I and 5′∆RNA I-dsRED mRNA (Figure 1B). Expression levels of dsRED protein were congruent with the efficiency of 5′dsRED mRNA delivery (Figure 1B). We obtained analogous results using another mRNA (GFP) and a different cell line (HepG2) (Figure S1). These results indicate that AuNP-DNA efficiently delivers mRNA into human cells, and that this delivered mRNA is capable of directing synthesis of targeted proteins.

### Influence of directionality of mRNA binding to AuNP-DNA conjugates and the presence of a cap structure at the 5′-terminus of mRNA on translation efficiency

**Figure 2:**
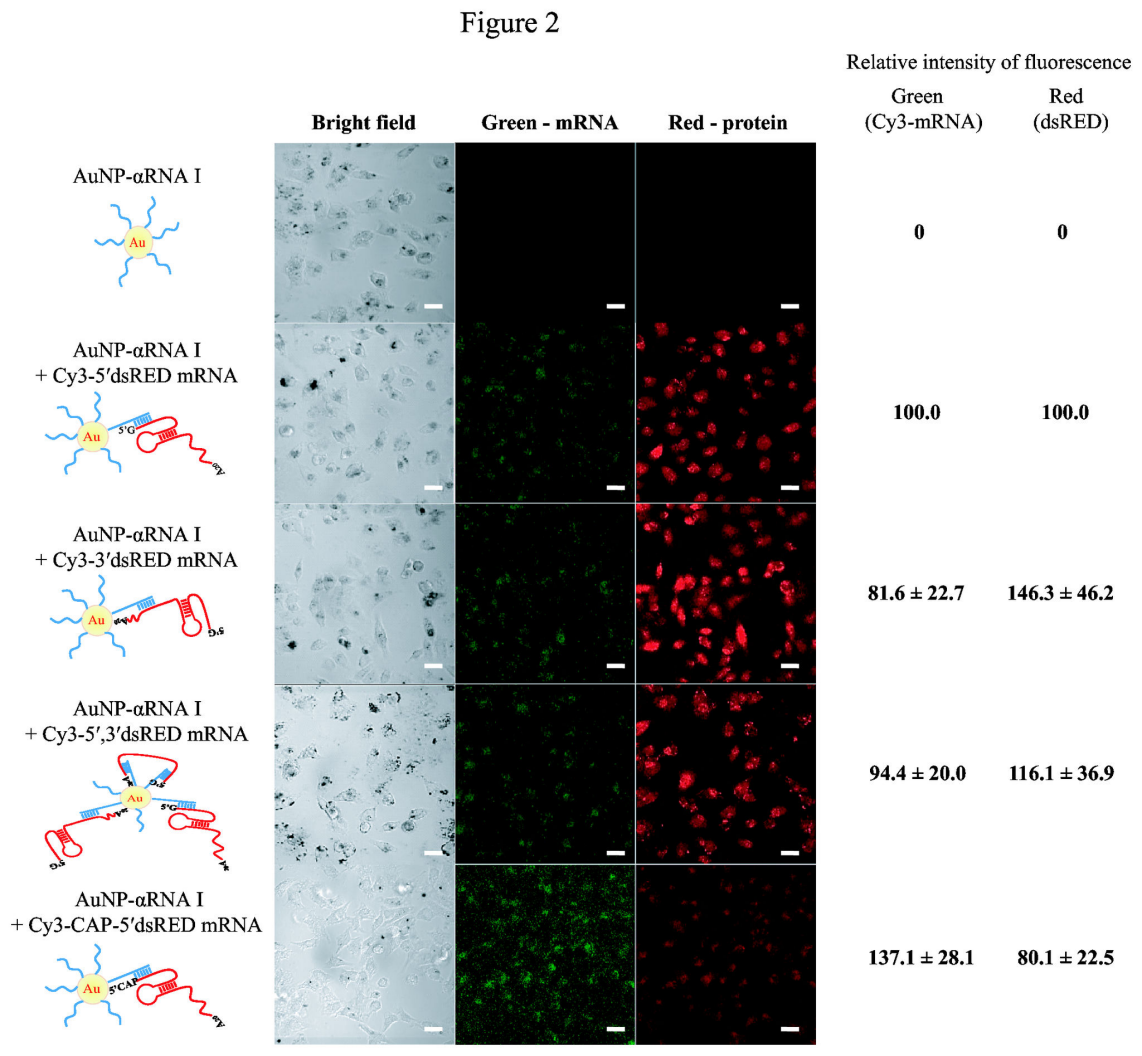
Effects of directionality of mRNA binding to AuNP-DNA and presence of cap structure at the 5′-terminus of mRNA. HeLa cells were treated with AuNP-αRNA I hybridized to Cy3-5’dsRED mRNA, Cy3-3’dsRED mRNA, Cy3-5′, 3’dsRED mRNA, or Cy3-CAP-5’dsRED mRNA. Fluorescent images show the Cy3-labeled mRNA signal in the left panels and the red fluorescent signal of the dsRED protein in the right panels. Representative confocal microscopy images of HeLa cells for each treatment are shown, along with schematic representations of *in* *vitro* transcripts and a diagram of the AuNP-αRNA I-mRNA binding structure. Bar indicates 20 µm. Relative intensities of fluorescence in cells were measured using ImageJ software. The values are shown by setting intensities of fluorescence from cells treated with AuNP-αRNA I + Cy3-5’dsRED mRNA as 100. Relative intensities of fluorescence were obtained from 10 cells of each treatment and are shown as the mean ± SEM (standard error of mean).

In order to assess the effects of directionality of mRNA binding to AuNP-DNA conjugates on translation efficiency, we designed and synthesized three different Cy3-labeled dsRED mRNA transcripts containing a sequence complementary to αRNA I at the 5′-, 3′-, or both 5′- and 3′-termini of mRNA (designated as Cy3-5′, Cy3-3′, or Cy3-5′,3′ dsRED mRNA, respectively). The directionality of mRNA binding to AuNP-DNA conjugates did not significantly affect the loading capacity of AuNP-αRNA I for these dsRED mRNAs (Figure S2). When HeLa cells were treated with AuNP-αRNA I loaded with these dsRED mRNAs, all showed similar mRNA transfection efficiency and protein expression levels (Figure 2).

A cap structure [m^7^G(5′) ppp(5′) G] at the 5′-end of eukaryotic mRNA has been shown to enhance translation [25]. It was also reported that the cap structure enhances translation of mRNA delivered by liposome-based transfection [4]. For these reasons, we examined translational efficiency in the presence of a cap structure at the 5′-end of synthetic mRNA. Cy3-labeled and 5′-capped dsRED mRNA that contained a sequence complementary to αRNA I at the 5′-terminus (Cy3-CAP 5'dsRED mRNA) was synthesized and delivered to HeLa cells via AuNP-αRNA I. As shown in Figure 2, the addition of a 5′-cap structure effected no significant changes in efficiency of mRNA delivery or protein expression levels. Based on these results, we used uncapped synthetic mRNAs containing a sequence complementary to αRNA I at the 5'-terminus in our remaining experiments.

### Ability of AuNP-DNA conjugates to deliver mRNA into xenograft tumors generated in mice

To test the potential of AuNP-DNA conjugates for therapeutic application, we extended our work to an *in vivo* xenograft tumor model. We chose *BAX* mRNA, which encodes a member of the BCL-2 family protein BAX (BCL-2-associated X-protein), a major mediator of apoptosis that initiates mitochondrial membrane permeabilization and pore formation, leading to the activation of the downstream apoptosis signaling pathway (for a recent review, see 26). These properties of BAX protein make it a sound candidate for cancer therapy. The synthetic *BAX* mRNA was hybridized with AuNP-αRNA I and delivered into MEF cells derived from a *Bax*-knockout mouse. The expression of BAX protein was detected in immunoblotting analysis using an anti-BAX antibody. As shown in Figure S3, the delivered AuNP-αRNA I-5’BAX mRNA was well-translated to BAX protein, while no fluorescence signal was observed in cells treated with AuNP-αRNA I alone. The *BAX* mRNA delivered by lipofectamine also effected efficient expression of BAX protein. The morphology of the transfected cells was altered, however, probably due to the cytotoxicity of lipofectamine in MEF cells (Figure S3).

**Figure 3:**
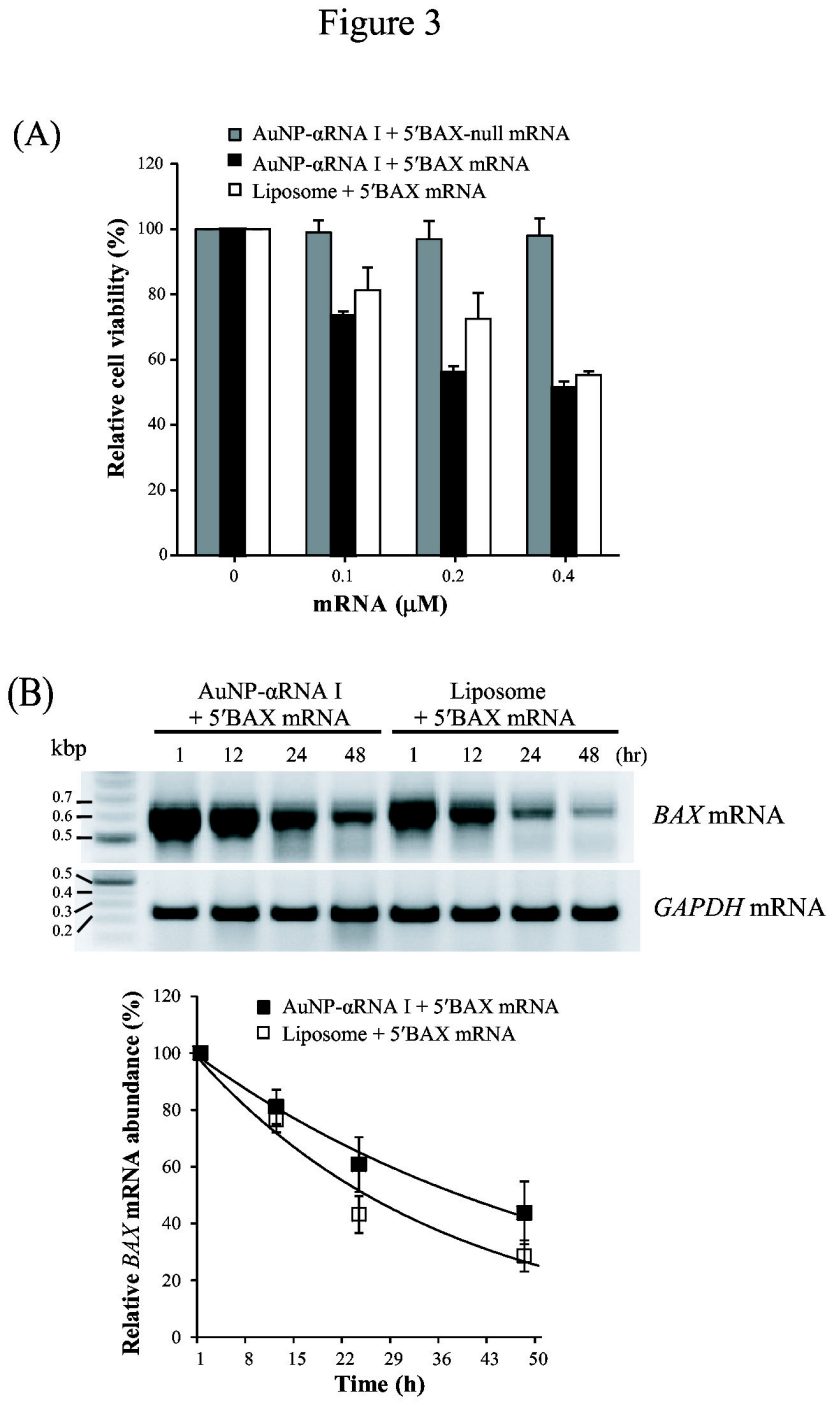
Influence of *BAX* mRNA delivery via AuNP-DNA conjugate or liposome on cell viability and mRNA half-life. (A) Relative viability of 5′BAX mRNA-transfected cells. HeLa cells were incubated with AuNP-αRNA I-5’BAX-null mRNA (Bax-null mRNA), AuNP-αRNA I-5’BAX mRNA, or liposome for 24 h. (B) RT-PCR analysis of 5′BAX mRNA to measure mRNA half-life. Amplification of cDNA from 5′BAX mRNA was achieved using a PCR primer (RNA I-AUG), designed specifically to bind to the 5’ UTR of 5′BAX mRNA, which is complementary to the cargo DNA of the AuNP-DNA. Equal loading was ensured using an internal control (GAPDH). The values were normalized against control cell values and are shown as the mean ± SEM (standard error of mean). Experiments were performed in triplicate and repeated at least three times.

We also tested whether composites of AuNP-αRNA I and *BAX* mRNA could be delivered to HeLa cells to induce apoptosis through BAX protein production. Induction of BAX-induced apoptotic cell death was assessed by measuring HeLa cell viability after incubation with AuNP-αRNA I-5’BAX-null mRNA or AuNP-αRNA I-5’BAX mRNA with varying mRNA concentrations. The BAX-null mRNA substitutes the start codon with a nonsense codon, preventing the production of functional full-length BAX protein. We found that cell viability was decreased by approximately 50% when the cells were treated with AuNP-αRNA I-5’BAX mRNA conjugates containing 0.2–0.4 µM *BAX* mRNA (Figure 3A). We observed a moderately decreased effect on cell viability when *BAX* mRNA was delivered by lipofectamine. When the cells were treated with AuNP-αRNA I-5’BAX-null mRNA conjugates, viability was unaffected. Semi-quantitative RT-PCR analysis of exogenously introduced *BAX* mRNA indicated that the half-life of the mRNA was approximately 16 h when delivered by AuNP-αRNA I (Figure 3B). The half-life of *BAX* mRNA was shorter (10 h) following lipofectamine-mediated delivery.

**Figure 4:**
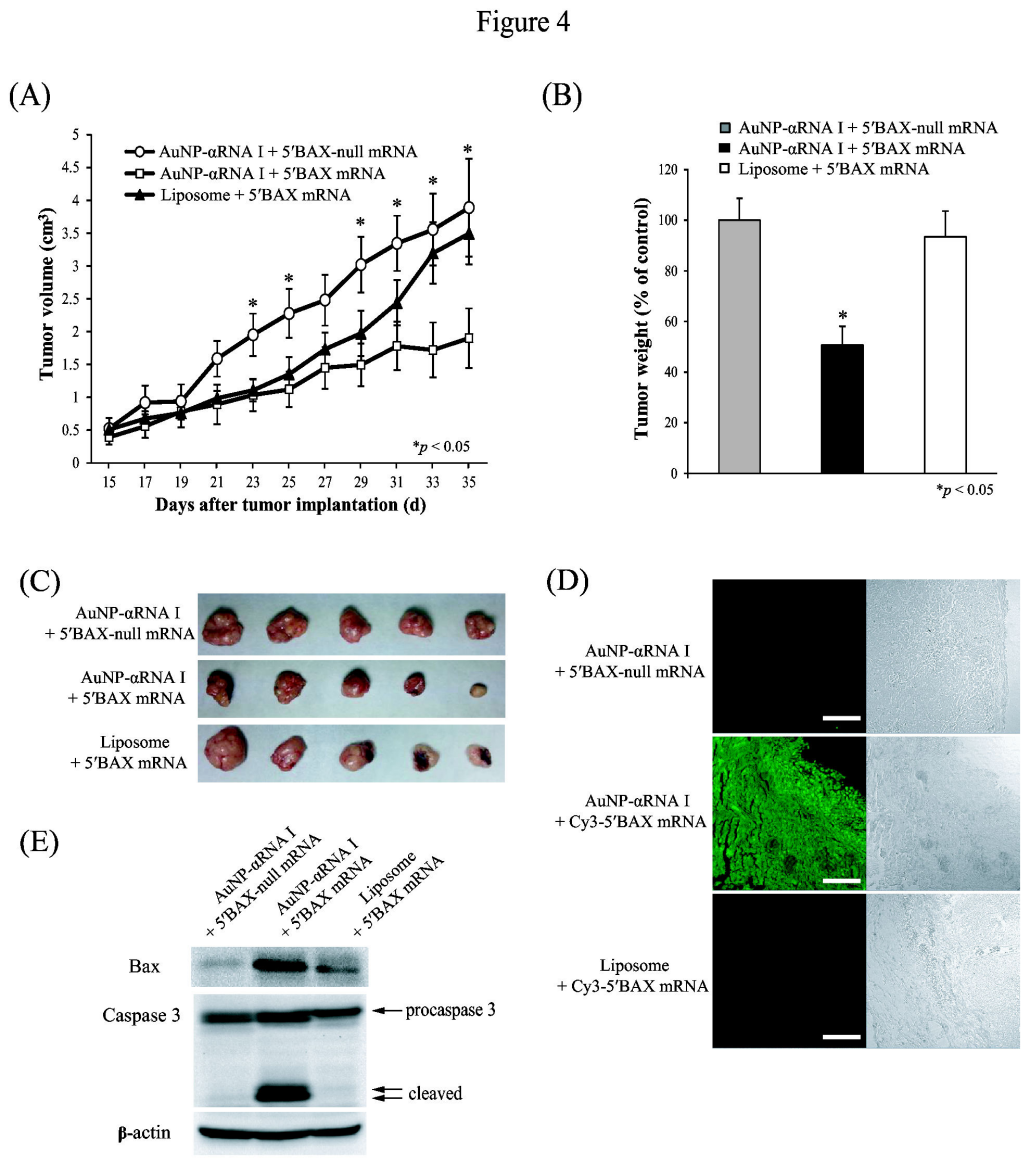
Retardation of tumor growth by delivery of AuNP-conjugated *BAX* mRNA. (A) The volume (mm^3^) of the tumor ((length × width^2^ × π)/6) was determined over a five-week period after xenograft implantation followed by injection of 5 nM AuNP-αRNA I-5’BAX mRNA in mice. AuNP-αRNA I-5’BAX-null mRNA and liposome-5'BAX mRNA were used as controls. Data (*n* = 8) are presented as the mean ± SEM, and asterisks indicate statistically significant values as compared to the corresponding controls (^*^ *P* < 0.05). (B) Tumor weight was measured at the time of sacrifice (30 days after implantation) and is presented as the % of control. (C) Actual sizes of representative tumors are shown. (D) Effective delivery of Cy3-labeled 5′BAX mRNA by AuNP-DNA conjugates into tumor tissue. Green signals indicate Cy3-5’BAX mRNA. Bar indicates 200 µm. (E) Western blot analysis of the tissue lysates was performed using anti-BAX, anti-Caspase 3, and anti-β-actin antibodies.

Based on these results, we examined whether AuNP-DNA conjugates could be used to deliver *BAX* mRNA into xenograft tumors in mice. HeLa cells were subcutaneously injected into the abdominal walls of BALB/c nu/nu immunodeficient mice to induce xenograft tumor development. Fourteen days after tumor cell implantation, AuNP-αRNA I-5’BAX-null mRNA, AuNP-αRNA I-5’BAX mRNA, or a mixture of lipofectamine and *BAX* mRNA was subcutaneously injected into the tumors every two days for 21 days. Consistent with *in vitro* cytotoxicity results, the volume (mm^3^) of xenografted tumors injected with the AuNP-αRNA I-Cy3-5′BAX mRNA conjugate was decreased significantly to 40% of the tumor volume in controls treated with AuNP-αRNA I-Cy3-5′BAX-null mRNA (Figure 4A). Representative tumors for each group are shown in Figure 4C; the weight of tumors injected with AuNP-αRNA I-Cy3-5′BAX mRNA was reduced to 50% of the control weights, as shown in Figure 4B.

Delivery of *BAX* mRNA by lipofectamine did not significantly inhibit tumor growth. The delivery of AuNP-αRNA I-BAX mRNA conjugates into tumor tissues was confirmed by analyzing tumor sections prepared from mice injected with AuNP-αRNA I-Cy3-5'BAX mRNA. The Cy3-labeled *BAX* mRNA signal was detected in sections of tumors injected with AuNP-αRNA I-BAX mRNA, whereas no signal was detected in sections of tumors from mice injected with either a mixture of lipofectamine and Cy3-labeled *BAX* mRNA or with AuNP-αRNA I-5’BAX-null mRNA (Figure 4D). Western blot analysis of BAX proteins in the xenograft tumors indicated that treatment with AuNP-αRNA I-5’BAX mRNA led to a marked increase in quantity of BAX (~250% of the BAX-null mRNA control), leading to increased caspase 3 activation, which induced apoptotic cell death (Figure 4E). Our results indicate that the AuNP-DNA conjugate is an effective way to deliver functional mRNA into live animals, directing the overexpression of biologically competent proteins.

## Discussion

Since the pioneering work of Malone et al. [4], who successfully delivered mRNA into cells using liposomes, numerous investigators have attempted to utilize mRNA for gene therapy. However, the development of mRNA-based gene therapy, which has been exclusively dependent on liposome carriers, has been challenged by ineffective delivery into living systems (for recent reviews, see 3,27,28). In the current study, we presented a safe, efficient, and straightforward method of delivering mRNA into living systems through the use of AuNP-DNA conjugates, which served as universal carriers.

We demonstrated that AuNP-DNA conjugates can efficiently load and deliver mRNA into human cells both *in vitro* and into xenograft tumors in mice. We found that mRNA delivered by AuNP-DNA conjugates to cells exhibited greater longevity than mRNA delivered by a liposome-based agent (i.e., lipofectamine; Figure 3B). This may be because mRNA molecules hybridized to AuNP-DNA conjugates are protected from attack by cellular ribonucleases; indeed, a previous study found that DNA oligos conjugated to AuNP were resistant to degradation by cellular nucleases [29].

Another advantage conferred by our AuNP-DNA conjugate is that it can be efficiently translated without a cap structure, whereas a 5′-cap must be added to mRNA delivered by liposome-based agents [4]. This is likely because the AuNP-DNA conjugate serves as a functional substitute for the cap structure by tethering translational factors to the mRNA. Supporting this is a recent report demonstrating enhanced *in vitro* translation of AuNP-DNA conjugates bound to mRNA by non-specific adsorption of translation-related factors [30].

The use of AuNP-DNA conjugates to deliver *BAX* mRNA to xenograft tumors in mice allowed for the synthesis of biologically functional BAX protein, which inhibited tumor growth by inducing apoptosis (Figure 4). In contrast, lipofectamine-mediated *BAX* mRNA delivery produced only minimal effects (Figure 4). We observed that AuNP-DNA-mRNA composites tended to spread from the injection site. This observation, along with a recent report showing how AuNP-DNA conjugates and extracellular proteins are co-transported into cells [31], led us to hypothesize that composites of AuNP-DNA-mRNA and non-specific proteins entering a cell could be transported into its neighbors.

Corollary to this idea is that composites of AuNP-DNA-mRNA conjugates are stable enough for intercellular transport, whereas composites of liposomes and mRNA tend to disassemble within a cell. This may explain why liposomes and AuNP-DNA exhibit similar mRNA delivery efficiency to cells grown in monolayers *in vitro* but not to three-dimensional tumors *in vivo*. While the precise molecular mechanisms of efficient mRNA delivery to xenograft tumors via AuNP-DNA conjugates remain unidentified, our results suggest that these conjugates may serve as a platform for the development of safe, effective non-biological agents of mRNA delivery in animals, which remains a key challenge to the use of mRNA in human therapy.

## Supporting Information

Figure S1 **Delivery of the *GFP* mRNA into HepG2 cells by AuNP-DNA conjugates.** (A) Measurement of the loading capacity of AuNP-αRNA I for the 5′GFP mRNA. Ten µl of various concentrations (0.5, 1.0, and 2.0 µM) of *GFP* mRNA were loaded (lanes 1-3) to access the loading capacity of AuNP-DNA for *GFP* mRNA. (B) Efficient delivery of AuNP-αRNA I-Cy3-5 ′GFP mRNA into HapG2 cells. Cells were treated with AuNP-αRNA I only or AuNP-αRNA I-Cy3-5’GFP mRNA. The final concentrations of the AuNP-αRNA I and Cy3-5’GFP mRNA were 1 nM and 0.2 µM, respectively. Fluorescent images show the Cy3-labeled mRNA signal in the left panels. Bar indicates 20 µm.
(EPS)

Figure S2 **Measurement of the loading capacity of AuNP-αRNA I for the 5′dsRED, 3′dsRED, and 5′, 3′ dsRED mRNA.** Ten µl of various concentrations (0.5, 1.0, and 2.0 µM) of *dsRED* mRNA were loaded (lanes 1-3) to access the loading capacity of AuNP-DNA for *dsRED* mRNA transcripts that contained a sequence complementary to αRNA I at the 5′-, 3′-, or both 5′- and 3’-termini of mRNA (designated as Cy3-5′, Cy3-3′, or Cy3-5′,3′ dsRED mRNA, respectively).
(EPS)

Figure S3 **Delivery of the *BAX* mRNA into MEF cells by AuNP-DNA conjugates.** MEF cells were treated AuNP-αRNA I, AuNP-αRNA I-5’dsRED mRNA, or a mixture of liposome and 5′dsRED mRNA. Representative confocal microscopy images of MEF cells for each treatment are shown. Fluorescence images in the left panels show red fluorescence signal of BAX protein. Bar indicates 20 µm.
(EPS)
